# Refinement of TOA Localization with Sensor Position Uncertainty in Closed-Form

**DOI:** 10.3390/s20020390

**Published:** 2020-01-10

**Authors:** Yi Gan, Xunchao Cong, Yimao Sun

**Affiliations:** 1 The 10th Research Institute of CETC, Chengdu 610036, China; sweat_gan1980@163.com; 2 School of Information and Communication Engineering, University of Electronic Science and Technology of China, Chengdu 611731, China; yimaosun@std.uestc.edu.cn

**Keywords:** source localization, time of arrival (TOA), small sensor network, sensor position uncertainty, closed-form, error refined

## Abstract

The subject of localization has received great deal attention in the past decades. Although it is perhaps a well-studied problem, there is still room for improvement. Traditional localization methods usually assume the number of sensors is sufficient for providing desired performance. However, this assumption is not always satisfied in practice. This paper studies the time of arrival (TOA)-based source positioning in the presence of sensor position errors. An error refined solution is developed for reducing the mean-squared-error (MSE) and bias in small sensor network (the number of sensors is fewer) when the noise or error level is relatively large. The MSE performance is analyzed theoretically and validated by simulations. Analytical and numerical results show the proposed method attains the Cramér-Rao lower bound (CRLB). It outperforms the existing closed-form methods with slightly raising computation complexity, especially in the larger noise/error case.

## 1. Introduction

Location determination is one of the classical research fields in signal processing. With the rise of the fifth-generation communication system (5G), Internet of things, automatic pilot and unmanned aerial vehicle, localization continues to receive great attention [1,2,3,4]. Based on different type of measurements, the common localization methods estimate the source position using time of arrival (TOA), time difference of arrival (TDOA), angle of arrival (AOA), Doppler frequency, Fingerprint and received signal strength (RSS) [5,6,7,8,9,10,11,12,13]. To improving the accuracy further, a series of hybrid method is developed by combining several kinds of measurements [14,15,16]. Since TOA localization has the precision advantage in position estimate, particularly in indoor environments [17], it attracts much interest in the netted mono-static radar, multi-static radar and distributed multi-input multi-output (MIMO) system.

TOA localization is categorized into two classes: circular-TOA method and elliptic-TOA method [18]. The circular-TOA localization [19] is usually applied to estimate the emitter location whose signal is intercepted by passive sensors. The TOAs (or ranges if the propagation speed is known) determine circles, taking the sensor as the center, that trace out the possible source location. The elliptic-TOA localization [20,21] creates ellipse loci through the signals traveling times from transmitters to the target and reflected back to received sensors. The ellipses from different sensor pairs intersect, yielding an estimate of the target position. In this paper, we focus our study on the circular-TOA case.

Time synchronization error is one of the factors that degrade the TOA localization performance. An opportunistic positioning method is proposed, exploiting radio transmitters and GPS-equipped nodes jointly [22]. G. Wang [23] considers the asynchronous sensors that the start transmission time is unknown. The source location is solved through second-order cone relaxation (SOCR). Considering the synchronization and localization problem simultaneously, the cooperation improves localization performance significantly without the requirement of high anchor node densities [24]. Y. Zou [25] presents an improved semidefinite programming (SDP) method when both asynchronism and sensor uncertainty occurs. Transforming the TOA measurements into TDOA measurements may be an effective way to eradicate the clock offset of the target’s clock [26]. Using the *a priori* of the existing unknown clock skew, the source position is solved by a fractional programming problem, resulting in the superior performance of the state-of-the-art methods. Y. Kang [27] proposes a technique to eliminate the time synchronization error iteratively, achieving a position estimator almost without time asynchronization. To reduce the computational cost, by applying robust squared-range (R-SR) and weighted least squares (WLS) criteria, [28] converts the originally nonconvex problem into a generalized trust region subproblem (GTRS) framework. It realizes comparable performance to the current methods with significantly higher effectiveness. To deal with multiple types of errors, the artificial neural network (ANN) and radial basis function (RBF) neural network are introduced to alleviate the accuracy loss due to the measurement error, non-line of sight (NLOS) error, and synchronization error [29].

Another factor that reduces the position estimation accuracy is sensor position uncertainty. Many works of literature study the source location estimate problem without considering the sensor position errors [30]. The research in [31] discusses the effect of sensor position errors. It indicates that using statistical knowledge of the sensor position errors is able to achieve the estimator with optimum localization performance. Commonly, there are two ways to take advantage of this statistical knowledge. On the one hand, the errors are estimated in terms of the coarse source position obtained under the assumption that sensor positions are precise. After calibrating the sensors, a more accurate source position estimate is achieved. This method is more efficient for locating multiple disjoint sources [32]. On the other hand, the sensor position error can be equivalently transformed into the measurement noise, where a new weighting matrix is constructed to cancel out the effect of sensor errors [33,34,35].

Usually, the Maximum Likelihood (ML) approaches which perform exhaustive grid search or iteration, and the SDP-based solutions are computation-cumbersome. Due to the low computation cost and easy implementation in engineering, the closed-form approaches are more attractive. The two-stage method, which is perhaps the most classical closed-form solution, resorts to weighted least squares (WLS) only [34]. The projection method for TOA localization is firstly proposed by [36]. The work in [37] improves the previous study and derives a closed-form projection method in reaching the CRLB performance. The improved projection method (IPM) doesn’t introduce a redundant variable, so it performs better than the two-step WLS (2WLS) [34]. However, the new projection matrix is not appropriate for some geometries. The research in [38] extends the multidimensional scaling (MDS) method [30] to the case with sensor position uncertainties. The modified MDS method implements better performance than the 2WLS when the sensor position errors are significant. However, the accuracy when using a small wireless sensor network, in which the number of sensors is limited, is poor. The recent research in [39] improves the TDOA localization performance in this situation. To the best of our knowledge, there is no such research on TOA localization in the presence of sensor position errors. The work in [40] illustrates that reducing the bias can improve the performance of the 2WLS method significantly. Therefore, subtracting the estimation bias from the solution in the second stage resulting in a better solution when the noise is relatively large. Since the bias is from the first stage which introduces the redundant variable, eliminating the redundant variable before estimating the bias is more effective, especially when the sensor network consists of fewer sensors.

This paper focuses on improving the TOA localization performance when the sensor network consists of fewer sensors. Converting the sensor position errors and measurement noise together as equivalent measurement noise, the TOA-based source location estimation is formulated as a weighted optimization problem with constraint. The proposed closed-form solution deals with this problem through three stages. The first stage introduces a weighting matrix in terms of the sensor position statistical knowledge to obtain a coarse solution, which is similar to the spherical-interpolation [14], where the constraint is neglected. The covariance of the coarse solution is derived. Then, the solution is improved through the relationship between the source position and redundant variable, using the covariance of stage-1. Stage three recalls the constraint to evaluate the estimate bias in the previous stage. The source position is refined by subtracting the estimated value, resulting in the final estimator. The analytical result shows the proposed method has CRLB performance when the noise and errors are mild. The simulation part verifies the theoretical analysis. It demonstrates that the new method outperforms the compared counterparts when the noise/error level is relatively higher in a small sensor network. Moreover, the mean-squared-error (MSE) and bias are comparable to the existing methods when using more sensors.

The main contributions of this paper are:

1. The weighted spherical-interpolation is derived, which solves the source position and redundant variable successively;
2. An approximate expression of the theoretical covariance analysis is presented for the weighted spherical-interpolation;
3. Eliminating the redundant variable, a refinement for the solution is proposed to improve the MSE and bias further;
4. The simulation shows the proposed method performs better than the state-of-the-art methods when using fewer sensors.

The novelties of this paper are:

1. Introducing a weighting matrix for spherical-interpolation resulting in the weighted spherical-interpolation;
2. Analyzing the covariance in the small noise region;
3. Refining the solution to improve the MSE and lower the bias further when using only 4 sensors.

This paper is organized as follows: Section 2 formulates the problem and clarifies the task. The proposed estimator is developed in Section 3. Section 4 presents the CRLB and theoretical performance analysis. Section 5 demonstrates the numerical results to show the advantages of the proposed when using fewer sensors. The conclusion is drawn in Section 6.

The unified notations in this paper is as following: Bold upper and lower case letter/symbol denote the matrix and vector. The vectors in this paper are column vectors. $x^{o}$ is the true value of x. ∥x∥ represents the Euclidean norm of x. diag{x} is a diagonal matrix consisted of the elements of x. $X^{T}$ and $X^{-1}$ are the transpose and inverse of X. x(i:j) denotes a subvector constructed by the *i*-th to *j*-th elements. ⊙ is the Hadamard product. sgn is the the signum operation.

## 2. Problem Formulation

Consider a scenario with *M* sensors deployed in a *N*-dimension space (N=2 or 3), whose true positions are $s_{i}^{o}$, i=1,2,⋯,M. Each sensor has the ability to measure the TOA from the unknown source. Denote the true value of the source position is $u^{o}$. The TOA between the unknown source and sensor *i* is

(1) $$\tau_{i}^{o}=∥u^{o}-s_{i}^{o}∥/c\;,$$

where *c* is the propagation speed. Multiplying *c* on both sides of (1) gives

(2) $$r_{i}^{o}=∥u^{o}-s_{i}^{o}∥\;,$$

where $r_{i}^{o}=c\tau_{i}^{o}$ is called range of arrival (ROA) measurement. In practice, the measured ROA is expressed as the true value with noise,

(3) $$r_{i}=r_{i}^{o}+n_{i}$$

where $n_{i}$ is the measurement noise. Collecting all ROAs together yields the measurement vector

(4) $$r=r^{o}+n\;,$$

where $r^{o}={\left[r_{1}^{o},r_{2}^{o},\cdots ,r_{M}^{o}\right]}^{T}$, n is modeled zero-mean with Gaussian distribution, and the covariance matrix of n is $Q_{r}$.

Noting the true sensor position is unavailable, only the erroneous counterpart is known,

(5) $$s_{i}=s_{i}^{o}+\Delta s_{i}\;.$$

The error vector $\Delta s={\left[\Delta s_{1}^{T},\Delta s_{2}^{T},\cdots ,\Delta s_{M}^{T}\right]}^{T}$ is Gaussian distribution with zero mean and covariance $Q_{s}$. We further assume that Δs is independent of the measurement noise n, and Δs is irrelevant across axis and sensors.

**Figure 1:**
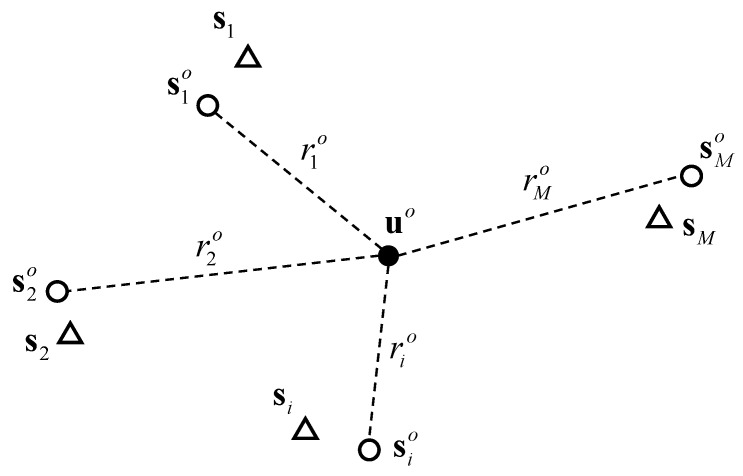
Localization scenario. Open circles are the unknown true sensor positions and open triangles represent the available imprecise sensor positions.

The localization scenario is shown in Figure 1. Our mission is locating the unknown source using the noisy TOA measurements and erroneous sensor positions, where the solution is closed-form. The performance with fewer sensors shall be improved compared with the existing closed-form method.

## 3. Refined Estimator

The derivation begins with the ROA expression (2). Squaring both sides of (2) and substituting (3) and (5) into the result, we arrive

(6) $$\begin{array}{c} -r_{i}^{2}+s_{i}^{T}s_{i}-2s_{i}^{T}u^{o}+v^{o}\\ =2r_{i}^{o}n_{i}+n_{i}^{2}+2{\left(u^{o}-s_{i}^{o}\right)}^{T}\Delta s_{i}-\Delta s_{i}^{T}\Delta s_{i}\;, \end{array}$$

where $v^{o}=u^{oT}u^{o}$. Stacking (6) across i=1,2,⋯,M yields matrix form equation

(7) $$h_{1}-A\psi^{o}=\epsilon \;,$$

where the unknown is $\psi^{o}={\left[u^{oT},v^{o}\right]}^{T}$,

(8) $$h_{1}=\left[\begin{array}{c} -r_{1}^{2}+s_{1}^{T}s_{1}\\ -r_{2}^{2}+s_{2}^{T}s_{2}\\ \vdots \\ -r_{M}^{2}+s_{M}^{T}s_{M} \end{array}\right]\;,$$

(9) $$A={\left[\begin{array}{cccc} 2s_{1} & 2s_{2} & \cdots & 2s_{M}\\ 1 & 1 & \cdots & 1 \end{array}\right]}^{T}\;,$$

(10) $$\epsilon =Bn+C\Delta s+\epsilon^{\prime }\;,$$

and

(11) $$B=2\mathrm{diag}\{r^{o}\}\;,$$

(12) $$C=2\left[\begin{array}{ccc} {\left(u^{o}-s_{1}^{o}\right)}^{T} & & \\ & \ddots & \\ & & {\left(u^{o}-s_{M}^{o}\right)}^{T} \end{array}\right]\;,$$

(13) $$\epsilon^{\prime }=n⊙n-{\left[\begin{array}{c} \Delta s_{1}^{T}\Delta s_{1}\\ \Delta s_{2}^{T}\Delta s_{2}\\ \vdots \\ \Delta s_{M}^{T}\Delta s_{M} \end{array}\right]}^{T}$$

is the second order noise term. Based on the least square criterion, finding $u^{o}$ is solving the optimization problem

(14) $$\min_{\psi }\;{\left(h_{1}-A\psi \right)}^{T}W\left(h_{1}-A\psi \right)$$

(15) $$s.t.\;\psi \left(N+1\right)=\psi {\left(1:N\right)}^{T}\psi \left(1:N\right)\;.$$

W is the weighting matrix approximating by

(16) $$W=E{\left[\epsilon \epsilon^{T}\right]}^{-1}≃{\left(BQ_{r}B+CQ_{s}C^{T}\right)}^{-1}\;.$$

Traditional method [34] first minimizes (14) by ignoring the constraint. Then the constraint is utilized in the second stage to improve the accuracy. In this paper, we shall follow a similar step to obtain the initial solution, but the processing is different from the 2-stage WLS (2WLS) [34]. A further stage is needed to refine the bias resulting in the final solution.

### 3.1. Coarse Solution

Rewriting (7) as

(17) $$h_{1}-G_{1}u^{o}+zv^{o}≃Bn+C\Delta s$$

where $G_{1}=A\left(:,1:N\right)$ and $z=1_{M}$. The second-order noise term $\epsilon^{\prime }$ is neglected. The existing sphere interpolation (SI) method [14] is suboptimal since it doesn’t take the different error in each row of (7) into account. We introduce a weighting matrix W that is set equal to $E{\left[\epsilon \epsilon^{T}\right]}^{-1}$ to balance the error in each element of e. Using such a W leads to the weighted sphere interpolation, reducing the covariance in the estimated ψ [41]. The optimization problem in (14) and (15) becomes

(18) $$\min_{u,v}\;{\left(h_{1}-G_{1}u+zv\right)}^{T}W\left(h_{1}-G_{1}u+zv\right)$$

(19) $$s.t.\;v=u^{T}u\;.$$

We now turn to solving the constrained optimization problem (18) and (19). If the constraint between u and *v* is ignored, the WLS solution of $u^{o}$ in terms of *v* is

(20) $$u=H(h_{1}+zv)\;,$$

where

(21) $$H={\left(G_{1}^{T}WG_{1}\right)}^{-1}G_{1}^{T}W\;.$$

Putting (20) into the right side of (17) yields

(22) $$h_{1}-G_{1}u+zv=R\left(h_{1}+zv^{o}\right)\;,$$

where $R=I_{M}+G_{1}H$. Using (22) to the original cost function (18), the optimization problem given by (18) and (19) is equivalent to

(23) $$\min_{v}\;{\left(h_{1}+zv\right)}^{T}R^{T}WR\left(h_{1}+zv\right)\;,$$

subject to (19). Ignoring the constraint (19), finding the suboptimal *v* is solving the unconstrained optimization problem (23). Taking the derivative of (23) with respect to *v* and equaling the result to zero, the estimation of $v^{o}$ is given by the WLS technology

(24) $$v=-{\left(z^{T}R^{T}WRz\right)}^{-1}z^{T}R^{T}WRh_{1}\;.$$

To obtain the source location estimate, the value of *v* calculated from (24) is substituted to (20).

### 3.2. Error Reduction

We next explore the constraint to reduce the error of the coarse u and *v* given by (20) and (24). Before formulating the problem in this stage, the covariance expression of u and *v* shall be detailed. To the best knowledge of the authors, there is no such analysis for weighted spherical-interpolation.

Subtracting both sides of (20) by the true value $u^{o}$ yields

(25) $$\Delta u=H(h_{1}+zv-G_{1}u^{o})\;.$$

Using the measurement Equation (17), $G_{1}u^{o}$ can be expressed by

(26) $$G_{1}u^{o}=h_{1}+zv^{o}-Bn-C\Delta s\;.$$

Substituting (26) to (25) and using $\Delta v=v-v^{o}$ yield

(27) Δu=H(zΔv+Bn+CΔs).

Δv is obtained by subtracting both sides of *v* (24) by the true value

(28) $$\begin{array}{cc} \Delta v & =-{\left(z^{T}R^{T}WRz\right)}^{-1}z^{T}R^{T}WR\left(h_{1}+zv^{o}\right)\\ & =\rho^{T}\left(Bn+C\Delta s\right)\;, \end{array}$$

where

(29) $$\rho =-{\left(z^{T}R^{T}WRz\right)}^{-1}R^{T}WRz\;.$$

Inserting (28) into (25) yields the expression of Δu,

(30) Δu=P(Bn+CΔs)

where

(31) $$P=H(I_{M}+z\rho^{T})\;.$$

Denoting $\Delta \psi ={\left[\Delta u^{T},\Delta v\right]}^{T}$, the covariance is

(32) $$\begin{array}{cc} \mathrm{cov}(\psi ) & =E[\Delta \psi \Delta \psi^{T}]\\ & =E\left\{\left[\begin{array}{cc} \Delta u\Delta u^{T} & \Delta u\Delta v\\ \Delta v\Delta u^{T} & \Delta v^{2} \end{array}\right]\right\}\\ & =\left[\begin{array}{cc} PW^{-1}P^{T} & PW^{-1}\rho \\ \rho^{T}W^{-1}P^{T} & \rho^{T}W^{-1}\rho \end{array}\right]\;. \end{array}$$

To reduce the errors in the coarse solution, a new formula shall be established in terms of the constraint (19). Recall the relationship between the source position $u^{o}$ and the residual variable $v^{o}$,

(33) $$v^{o}=u^{oT}u^{o}\;.$$

Commonly, it is reasonable to consider the errors in the estimated unknown parameters, e.g., Δu and Δv, as additional [33,34,40,42]. Thus, we can express the redundant variable *v* as

(34) $$v=v^{o}+\Delta v\;.$$

Substituting (33) to (34) results

(35) $$v-u^{oT}u^{o}=\Delta v\;.$$

Squaring both sides of

(36) $$u=u^{o}+\Delta u$$

elementwise results in

(37) $$u⊙u-u^{o}⊙u^{o}=2u^{o}⊙\Delta u\;,$$

where the second-order term of Δu is dropped. Together with the equation obtained in (35), we have

(38) $$h_{1}^{\prime }-G_{1}^{\prime }\psi^{\prime o}=B^{\prime o}\Delta \psi \;,$$

where

(39) $$\psi^{\prime o}=u^{o}⊙u^{o}\;,$$

(40) $$h_{1}^{\prime }=\left[\begin{array}{c} u⊙u\\ v \end{array}\right]\;,$$

(41) $$G_{1}^{\prime }=\left[\begin{array}{c} I_{N}\\ 1_{N}^{T} \end{array}\right]\;,$$

(42) $$B^{\prime o}=\left[\begin{array}{cc} 2\mathrm{diag}\{u^{oT}\} & \\ & 1 \end{array}\right]\;.$$

Thus, the estimate of $\psi^{\prime o}$ is the one that satisfies

(43) $$\min_{\psi^{\prime }}\;{\left(h_{1}^{\prime }-\psi^{\prime }\right)}^{T}W^{\prime }\left(h_{1}^{\prime }-G_{1}^{\prime }\psi^{\prime }\right)\;,$$

which is given by

(44) $$\psi^{\prime }={\left(G_{1}^{\prime T}W^{\prime }G_{1}^{\prime }\right)}^{-1}G_{1}^{\prime T}W^{\prime }h_{1}^{\prime }\;,$$

where the weighting matrix $W^{\prime }$ is approximated as

(45) $$W^{\prime }={\left(B^{\prime }\mathrm{cov}\left(\psi \right)B^{\prime }\right)}^{-1}\;,$$

where $B^{\prime o}$ is replaced by $B^{\prime }$ using u instead. $W^{\prime }$ is related to W by (32).

The estimation of $u^{o}$ is the square root of $\psi^{\prime }$ in term of (39). However, there is sign ambiguity since using only $\psi^{\prime }$ can’t determine the sign of u elementwise. One way to solve this sign ambiguity is to use the sign given by the first stage [10]. Thus, the final error-reduced estimate is

(46) $$u^{\prime }=\mathrm{sgn}\left(u\right)⊙\sqrt{\psi^{\prime }}$$

where u is the result given in the first stage (20), and

(47) $$v^{\prime }=u^{\prime T}u^{\prime }$$

in terms of (33).

### 3.3. Refinement

The last stage will refine the solution above. Express the $u^{\prime }$ as

(48) $$u^{\prime }=u^{o}+\Delta u^{\prime }$$

where $\Delta u^{\prime }$ is the estimation error. So the expression of $v^{o}$ in terms of $u^{\prime }$ and $\Delta u^{\prime }$ is

(49) $$v^{o}={\left(u^{\prime }-\Delta u^{\prime }\right)}^{T}\left(u^{\prime }-\Delta u^{\prime }\right)\approx u^{\prime T}u^{\prime }-2u^{\prime T}\Delta u^{\prime }\;.$$

Substituting (48) and (49) to (17) and dropping the second order noise term yield

(50) $$h_{2}-G_{2}\Delta u^{\prime }≃Bn+C\Delta s\;,$$

where

(51) $$h_{2}=h_{1}-G_{1}u^{\prime }+zv^{\prime }\;,$$

(52) $$G_{2}=2{\left[\begin{array}{c} {\left(u^{\prime }-s_{1}\right)}^{T}\\ {\left(u^{\prime }-s_{2}\right)}^{T}\\ \vdots \\ {\left(u^{\prime }-s_{M}\right)}^{T} \end{array}\right]}^{T}\;.$$

The estimation of error Δu is

(53) $$\Delta {\hat{u}}^{\prime }={\left(G_{2}^{T}WG_{2}\right)}^{-1}G_{2}^{T}Wh_{2}\;.$$

The final solution is

(54) $$\hat{u}=u^{\prime }-\Delta {\hat{u}}^{\prime }\;.$$

**Remark** **1.** *The weighting matrix W is determined by the true value of the source position. A remedy is setting B as identity and $C=I_{M}⊗I_{N}/N$ for a coarse solution. Then, the weighting matrix can be updated through the coarse solution and a better initial solution result. In the refinement stage, the updated W is sufficient for an accurate final solution.*

**Remark** **2.** *The relationship *(33)* has been used in Section 3.2, but it is insufficient to obtain the best estimate. Normally, the constrained weighted least squares (CWLS) is better than the two-stage WLS [43,44,45,46], which means dealing with the quadratic cost function and the constraint subsequently is suboptimal. Although the 2WLS approaches the CRLB in small noise region, its performance degradation is distinct as the noise level increases. The work in [40] illustrates that the large bias is the reason for performance degradation. Reducing the bias of the first stage can improve performance significantly. Therefore, subtracting the estimation bias from the solution in the second stage can improve the performance when the noise is relatively large. Since the bias is from the first stage which introduces the redundant variable, eliminating the redundant variable before estimating the bias is more effective when the sensor network consists of fewer sensors.*

## 4. Analysis

### 4.1. CRLB

The CRLB is the most widely applied technique for bounding the ability to estimate the interest parameters from the given data. Although the CRLB of TOA localization when the sensor positions are uncertain has been developed in [34], we will present the CRLB from the other perspective for the theoretical performance comparison.

Equation (10) can be rewritten as

(55) $$\epsilon =B\tilde{n}\;,$$

where

(56) $$\tilde{n}=n+B^{-1}C\Delta s\;.$$

In (56), the sensor errors are linearly transformed as an additional term of measurement noise. Thus, $\tilde{n}$ is called the equivalent noise. This transformation combines the noise and sensor position errors, which simplifies the algorithm derivation and analysis expression.

In terms of the equivalent noise, the corresponding covariance matrix is

(57) $$Q=E\left[\tilde{n}{\tilde{n}}^{T}\right]=Q_{r}+B^{-1}CQ_{s}C^{T}B^{-1}\;.$$

Then, the CRLB is given by [41]

(58) $$\mathrm{CRLB}\left(u^{o}\right)={\left(\frac{\partial r^{oT}}{\partial u^{o}}Q\frac{\partial r^{o}}{\partial u^{oT}}\right)}^{-1}$$

where

(59) $$\frac{\partial r^{o}}{\partial u^{oT}}=\left[\begin{array}{c} {\left(u^{o}-s_{1}^{o}\right)}^{T}/r_{1}^{o}\\ {\left(u^{o}-s_{1}^{o}\right)}^{T}/r_{1}^{o}\\ \vdots \\ {\left(u^{o}-s_{M}^{o}\right)}^{T}/r_{M}^{o} \end{array}\right]\;.$$

### 4.2. Covariance

The estimator in the first and the second stage can be expressed as

(60) $$\begin{array}{cc} u^{\prime } & =u^{o}+\Delta u^{\prime }\;,\\ \Delta {\hat{u}}^{\prime } & =\Delta u^{\prime }+\delta u^{\prime }\;. \end{array}$$

Substituting (60) to (54) yields

(61) $$\hat{u}=u^{o}-\delta u^{\prime }\;.$$

Therefore, the final estimation error is −δu,

(62) $$\begin{array}{cc} -\delta u^{\prime } & =\hat{u}-u^{o}\\ & =-{\left(G_{2}^{T}WG_{2}\right)}^{-1}G_{2}^{T}W\left(Bn+C\Delta s\right)\;. \end{array}$$

After neglecting the noise/error terms higher than second order, the covariance of $\hat{u}$ is approximately

(63) $$\mathrm{cov}\left({\hat{u}}^{\prime }\right)=E{\left[\delta u^{\prime }\delta u^{\prime T}\right]}^{-1}≃{\left(G_{2}^{T}WG_{2}\right)}^{-1}.$$

### 4.3. Comparison

Under the small noise conditions

(64) $$\begin{array}{c} 1)\;n_{i}/r_{i}^{o}\approx 0\;,\\ 2)\;∥\Delta s_{i}∥/r_{i}^{o}\approx 0\;, \end{array}$$

Substituting (16) and (57) to (63), the inverse of the theoretical covariance of $\hat{u}$ is

(65) $$\mathrm{cov}{\left(\hat{u}\right)}^{-1}\approx G_{2}^{oT}B^{-1}QB^{-1}G_{2}^{o}\;.$$

For simplicity, we denote

(66) $$G_{3}^{o}=B^{-1}G_{2}^{o}\;.$$

Substituting (11) and (52) into (66), after some algebraic manipulations, and comparing the result with (59) yield

(67) $$G_{3}^{o}=\frac{\partial r^{o}}{\partial u^{oT}}\;.$$

Therefore, we conclude that (63) equals to the CRLB if the noise is mild.

## 5. Simulations

Simulations run with M=6 sensors. It is worth to note that the proposed method is applicable to both two-dimensional and three-dimensional cases. Only the results for the more complicated case of 3-D are illustrated in this Section. The certain sensor positions are listed in Table 1. We use a near source and a far source for the experiments: $u_{near}^{o}={\left[400,350,550\right]}^{T}$ m and $u_{far}^{o}={\left[2000,1750,2250\right]}^{T}$ m. The covariance matrix of TOAs and sensor positions are $Q_{r}=\sigma_{r}^{2}I_{M}$ and $Q_{s}=\sigma_{s}^{2}\mathrm{diag}\left\{\rho \right\}⊗I_{N}$, where ρ is a scaling vector for sensor position errors. The proposed method is compared with three closed-form solutions, 2WLS [34], improved projection method (IPM) [37] and multidimensional scaling (MDS) based method [30], and the maximum likelihood estimator (MLE) implemented through Gauss-Newton iteration. In addition, the CRLB is introduced as a benchmark. The number of ensemble runs is K=1000, unless stated otherwise. The performance is evaluated by MSE and bias,

(68) $$MSE(u)=\frac{1}{K}\sum_{k=1}^{K}{\left(u_{k}-u^{o}\right)}^{T}\left(u_{k}-u^{o}\right)\;,$$

(69) $$bias(u)=\frac{1}{K}\sum_{k=1}^{K}∥u_{k}-u^{o}∥\;,$$

where $u_{k}$ is the estimates at ensemble run *k*.

### 5.1. Fewer Sensors

**Figure 2:**
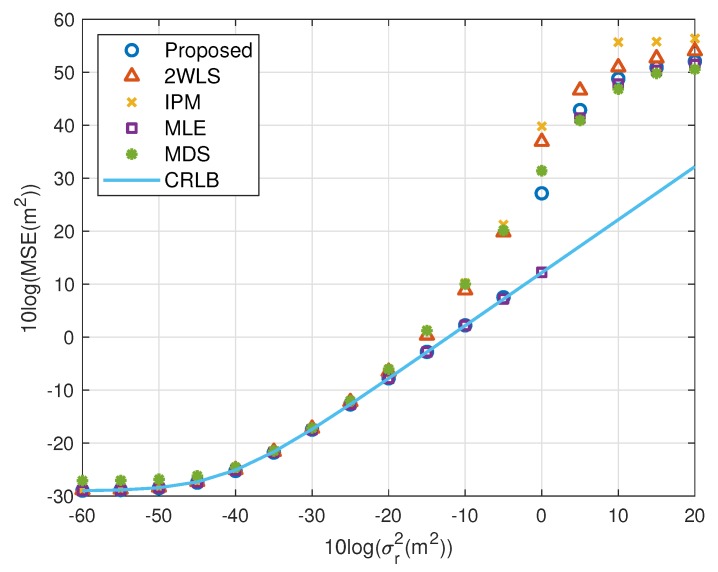
MSE performance as noise power increasing (near source, 4 sensors).

**Figure 3:**
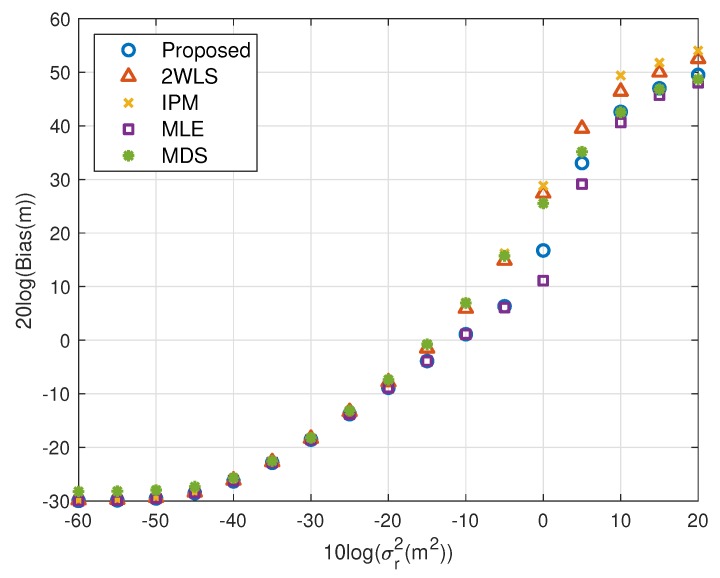
Bias performance as noise power increasing (near source, 4 sensors).

**Figure 4:**
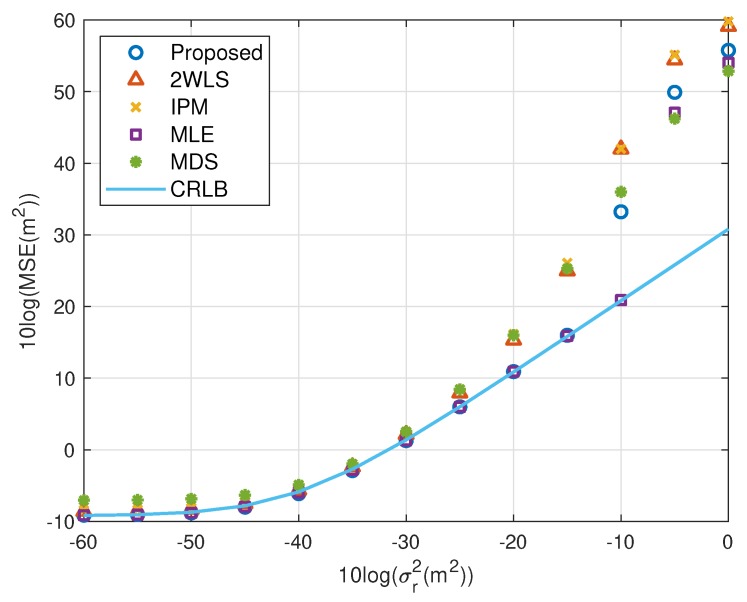
MSE performance as noise power increasing (far source, 4 sensors).

**Figure 5:**
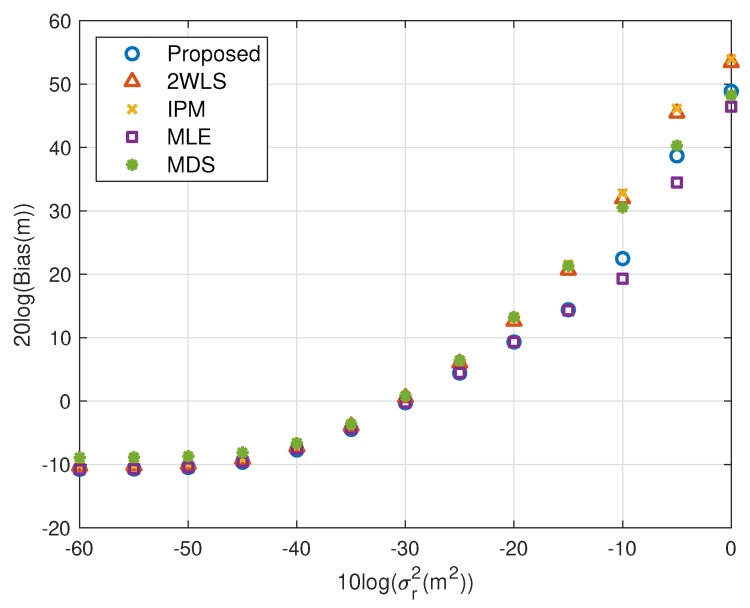
Bias performance as noise power increasing (far source, 4 sensors).

The motivation of this research is reducing the MSE and bias when using fewer sensors. Figure 2, Figure 3, Figure 4 and Figure 5 illustrate the MSE and bias as the TOA measurement noise increases, where the sensor network consist of 4 sensors, $s_{1}$, $s_{2}$, $s_{3}$ and $s_{4}$. The sensor position error level is fixed at $\sigma_{s}^{2}=10^{-5}$ m^2^. The scaling vector is $\rho ={\left[10,2,10,40\right]}^{T}$. For the near source, the 2WLS, IPM and the proposed algorithm have comparable MSE and bias if $\sigma_{r}^{2}⩽10^{-2}$ m^2^, while the MDS is more robust than 2WLS, IPM and even the proposed in relatively larger error region, although the sensor position errors are not taken into account. When the noise increases, the 2WLS, IPM and MDS deviate the CRLB after the noise power higher than $10^{-2}$ m^2^, while the proposed method diverges over 15 dB later than the 2WLS and IPM. If the noise increases further, the proposed method keeps the MLE-level MSE and bias even when the noise is significantly large. The MLE deviates the MLE after $\sigma_{r}^{2}>1$ m^2^. The result is similar for the far source, the proposed method deviates the CRLB when $\sigma_{r}^{2}>10^{-1.5}$ m^2^, while the performance of the 2WLS, IPM and MDS become worse at $\sigma_{r}^{2}=10^{-3}$ m^2^. The proposed method outperforms the 2WLS and IPM, even after deviating the CRLB. And the proposed algorithm lowers the bias, especially when the $\sigma_{s}^{2}>10^{-2}$ m^2^ for near target and $\sigma_{r}^{2}>10^{-3}$ m^2^ for far source. The MLE is the best but delays the threshold only 5 dB.

**Figure 6:**
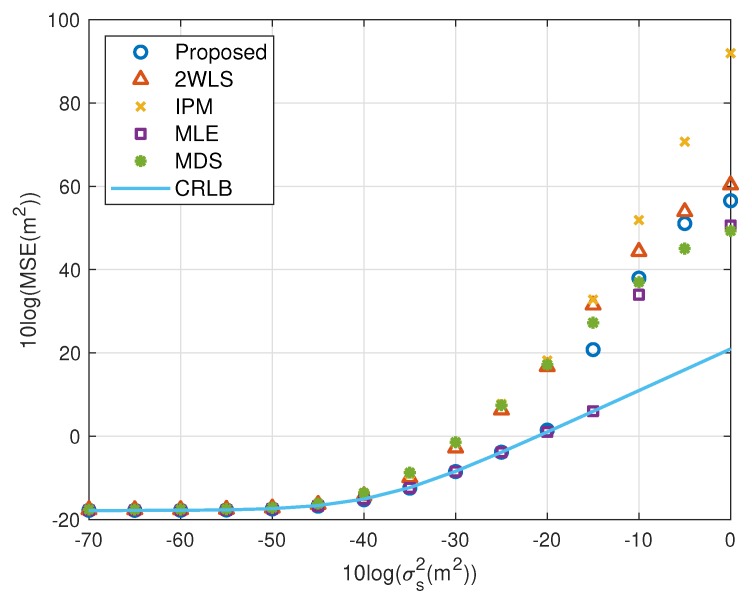
MSE performance as error level increasing (near source, 4 sensors).

**Figure 7:**
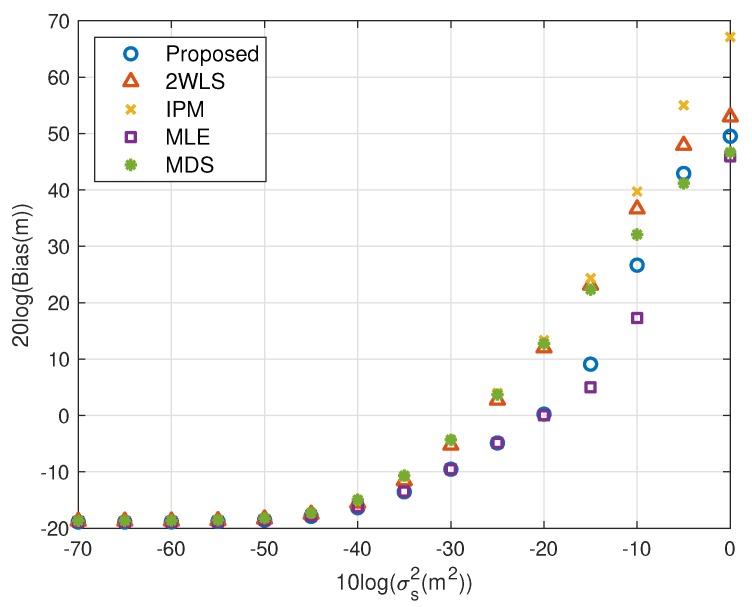
Bias performance as error level increasing (near source, 4 sensors).

**Figure 8:**
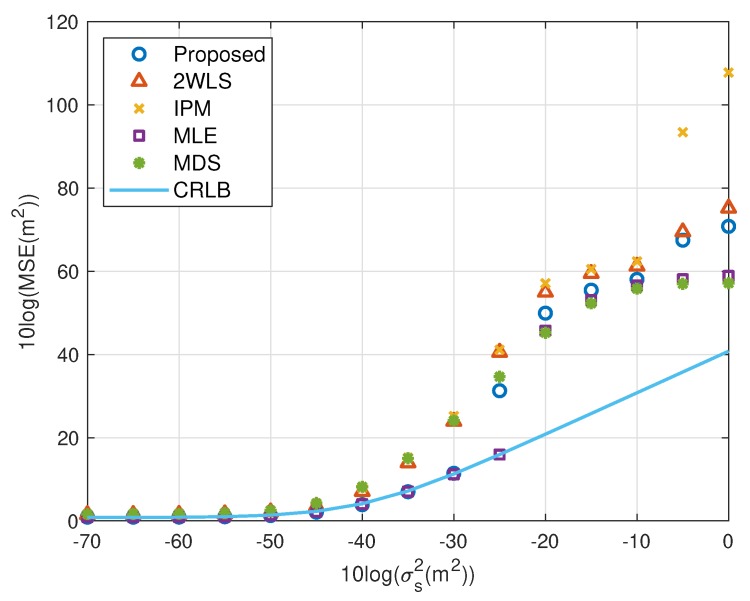
MSE performance as error level increasing (far source, 4 sensors).

**Figure 9:**
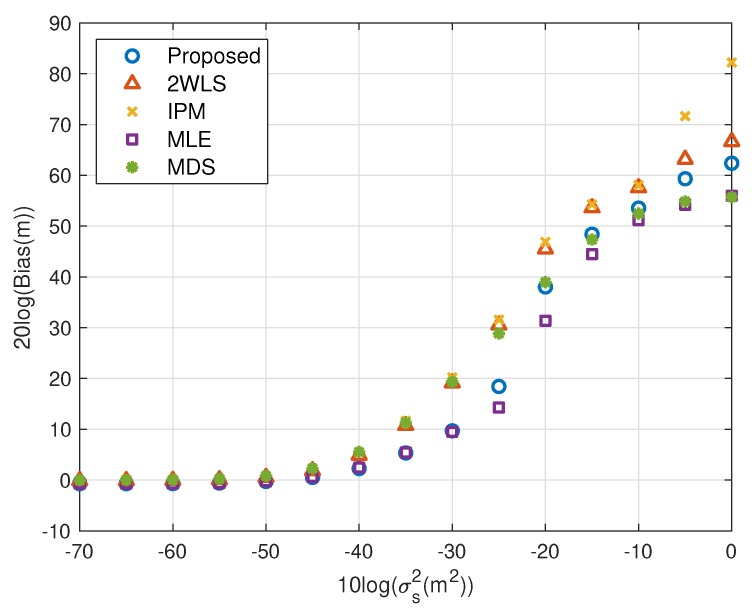
Bias performance as error level increasing (far source, 4 sensors).

The performance behavior as the position error level of sensors varies is demonstrated in Figure 6, Figure 7, Figure 8 and Figure 9. The sensors’ deployment is the same as the experiments above. The TOA measurement noise power is set as $\sigma_{r}^{2}=10^{-3}$ m^2^. If the source is near, Figure 6 indicates that the 2WLS, IPM and MDS fail to reach the CRLB after $\sigma_{s}^{2}>10^{-4}$ m^2^, $10^{-4}$ m^2^ and $10^{-4.5}$ m^2^ respectively, while the proposed method delays the divergence to $\sigma_{s}^{2}>10^{-2}$ m^2^. And the MLE delays the threshold 5 dB further. In Figure 7, five methods asymptotically have the same level bias in small error region. The proposed method can yield a much smaller bias about 15 dB around the error level at $\sigma_{s}^{2}>10^{-2}$ m^2^. It lowers the bias over 10 dB than the compared three closed-form methods at most, which is comparable to the MLE. Moreover, Figure 7 and Figure 9 imply the reason that 2WLS, IPM and MDS deviate the CRLB earlier. The raising bias deteriorates the MSE performance. The results when locating a far source are similar to before. The proposed achieves the CRLB accuracy until $\sigma_{s}^{2}=10^{-3}$ m^2^ and have bias less than that of the three existing closed-form methods since $\sigma_{s}^{2}>10^{-5}$ m^2^.

To show the effectiveness of the proposed work more clearly, the performance loss is presented with the percentage with respect to the CRLB. The results for near source and far source are listed in Table 2 and Table 3. The proposed method provides comparable MSE performance to the MLE before the divergence. The MSE of the proposed and MLE is very close to the CRLB where the difference is less than 2%. Noting the ensemble runs is limited, and the bias has been subtracted from the generated noise, the MSE may be slightly lower than the CRLB. 2WLS and IPM have larger performance loss with respect to the CRLB. The MDS is more robust than 2WLS, IPM and even the proposed in relatively larger error region, although the sensor position errors are not taken into account.

### 5.2. More Sensors

**Figure 10:**
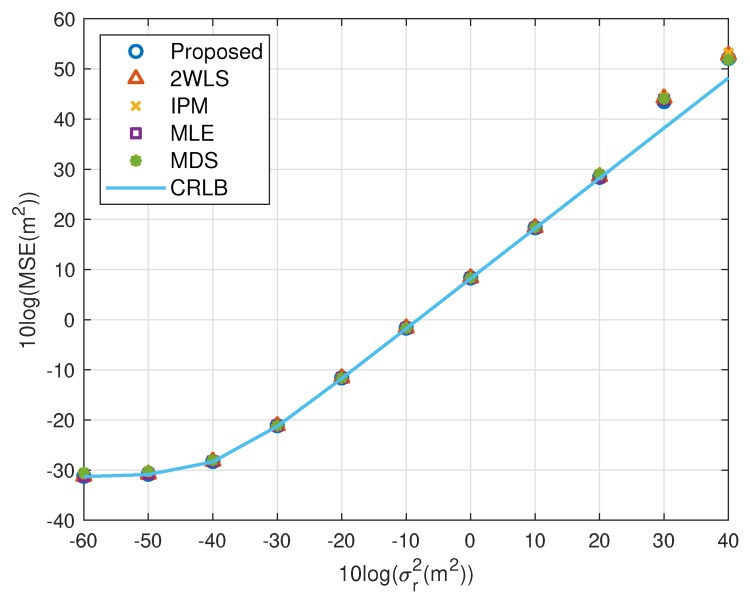
MSE performance as noise power increasing (near source, 6 sensors).

**Figure 11:**
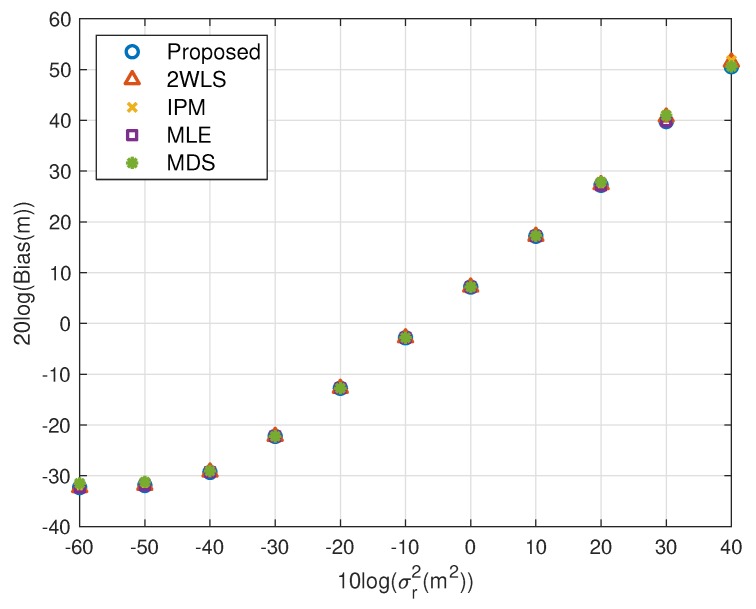
Bias performance as noise power increasing (near source, 6 sensors).

**Figure 12:**
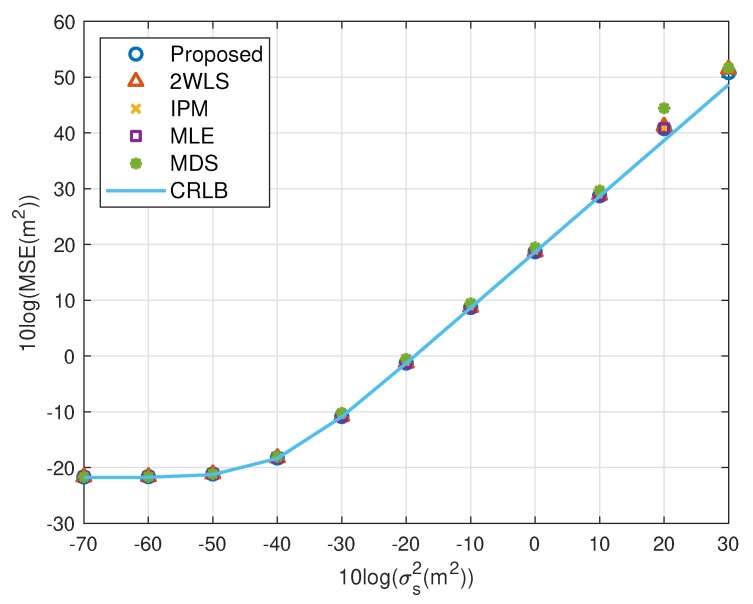
MSE performance as error level increasing (near source, 6 sensors).

**Figure 13:**
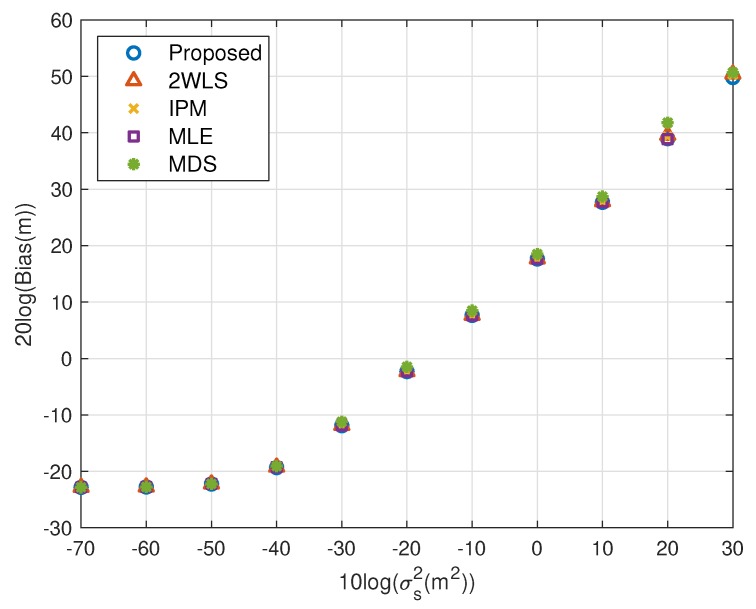
Bias performance as error level increasing (near source, 6 sensors).

The expectation of the proposed solution is improving the performance when using fewer sensors, while the accuracy under a normal sensor network with sufficient sensors should not be worse than the existing methods. The simulations above illustrate the advantages of the proposed closed-form solution on both MSE and bias. In this subsection, we’d like to show the new approach has comparable MSE and bias to the compared closed-form solutions. The scaling vector is $\rho ={\left[10,2,10,40,20,3\right]}^{T}$. To limit the number of illustrations, we only present the results for single near source in Figure 10, Figure 11, Figure 12 and Figure 13. They display similar performance of all methods as the measurement noise power or sensor error level increases, albeit the proposed is slightly better than the 2WLS, IPM and MDS when $\sigma_{r}^{2}>10^{2}$ m^2^ or $\sigma_{s}^{2}>10$ m^2^. The simulation results verify that the proposed method improves the performance when using 4 sensors but does as good as the existing methods when using more sensors, rather than designed for 4 sensors-case only.

### 5.3. Computation Time

The processing time of the proposed method and the compared counterparts is collected by Matlab 2018b on a typical PC with AMD R5 3500X CPU. The running time of 20000 independent tests is listed in Table 4. The proposed method is less computation-complexity than MLE, IPM and MDS and is comparable with the 2WLS method.

### 5.4. Summary of Simulations

We shall summarize the simulation results. The proposed method performs better than the existing methods because it gives a more accurate coarse location than IPM and refines the bias that 2WLS and MDS do not. The coarse solution of IPM can’t reach the CRLB in some cases, e.g., the configuration used in this paper. After refinement, the performance is still worse than the proposed. However, the coarse solution of the proposed is able to attain the CRLB when the noise is quite small, as well as the final solution of 2WLS and MDS. The refining step reduces bias further, as shown in Figure 3, Figure 5, Figure 7 and Figure 9. That is the reason why the proposed outperforms the 2WLS and MDS if the noise increases from 10^−2^ m^2^ to 1 m^2^ for a near source or from 10^−3^ m^2^ to 0.1 m^2^ for a far source.

The proposed method performs worse than MLE and MDS when the noise power is relatively high. MLE is implemented by the Gauss-Newton iteration, which is the best for parameter estimation. The MDS has been verified to be robust for the source localization. It has better MSE than the proposed method in larger noise region, but it is inferior to the proposed both MSE and bias in other cases.

Moreover, the proposed method is less computation-consumption but the performance is better than the existing in relatively large noise and error conditions.

## 6. Conclusions

A refined TOA localization solution is proposed in this paper, with better MSE and bias than the existing methods when the sensors composing the network are few. This new solution is closed-form, whose three stages take the WLS only. The MSE is analyzed theoretically, which is proven mathematically attaining the CRLB if the measurement noise and sensor errors are small. The MSE and bias are examined in the simulations, verifying the analytical result and illustrating the superiority of the proposed method.

Locating a source in a *N*-dimensional (*N*-D) space using TOA requires *N* sensors at least, which means the minimum sensor numbers for a *N*-D space target positioning is *N*. However, the proposed method, as well as the 2WLS and IPM, are not applicable when there are only *N* sensors since the redundant parameter is introduced for pseudo-linearization. The TOA localization using the minimal sensor network may be an interesting subject for further study.

## Figures and Tables

**Table 1:** Certain sensor positions.

Sensor	$s_{1}$	$s_{2}$	$s_{3}$	$s_{4}$	$s_{5}$	$s_{6}$
x (m)	−100	200	400	350	300	300
y (m)	100	−300	150	200	500	100
z (m)	−100	−200	100	100	200	150

**Table 2:** MSE increasing percentage (near source).

Noise Power (dB)	−60	−50	−40	−30	−20	−10	0	10	20
Proposed (%)	0.42	−1.42	−0.57	−1.62	0.34	1.10	3.04 × 10^3^	4.55 × 10^4^	9.61 × 10^4^
2WLS (%)	3.54	1.74	1.38	3.37	34.15	367.12	2.96 × 10^4^	7.55 × 10^4^	1.52 × 10^4^
IPM (%)	5.59	4.36	1.31	5.48	50.56	538.84	5.78 × 10^4^	2.23 × 10^5^	2.62 × 10^4^
MLE (%)	0.40	−1.22	−0.94	−1.38	0.34	1.05	1.27	3.60 × 10^4^	8.39 × 10^3^
MDS (%)	54.86	44.10	12.83	5.44	49.05	508.94	8.24 × 10^3^	2.89 × 10^4^	6.79 × 10^3^

**Table 3:** MSE increasing percentage (far source).

Noise Power (dB)	−60	−50	−40	−30	−20	−10	0
Proposed (%)	0.92	−1.84	−1.27	−1.06	0.93	1.64 × 10^3^	3.12 × 10^3^
2WLS (%)	14.34	10.58	6.43	19.37	178.63	1.30 × 10^4^	6.73 × 10^4^
IPM (%)	18.34	14.94	8.85	27.36	237.50	1.31 × 10^4^	7.90 × 10^4^
MLE (%)	0.86	−0.88	−1.32	−1.68	0.88	1.98	2.09 × 10^4^
MDS (%)	62.80	53.94	25.53	30.20	225.49	3.20 × 10^3^	1.59 × 10^4^

**Table 4:** Computation time.

Method	Proposed	2WLS	MLE	IPM	MDS
Time (s)	29.90	21.69	142.07	72.80	112.15
Rel. Time	1	0.73	4.75	2.43	3.75

## References

[1] Witrisal K., Meissner P., Leitinger E., Shen Y., Gustafson C., Tufvesson F., Haneda K., Dardari D., Molisch A.F., Conti A. (2016). High-Accuracy Localization for Assisted Living: 5G systems will turn multipath channels from foe to friend. IEEE Signal Process. Mag..

[2] Buehrer R.M., Wymeersch H., Vaghefi R.M. (2018). Collaborative Sensor Network Localization: Algorithms and Practical Issues. Proc. IEEE.

[3] Aditya S., Molisch A.F., Behairy H.M. (2018). A Survey on the Impact of Multipath on Wideband Time-of-Arrival Based Localization. Proc. IEEE.

[4] Zhang F., Sun Y., Wan Q. (2019). Calibrating the error from sensor position uncertainty in TDOA-AOA localization. Signal Process..

[5] Díez-González J., Álvarez R., Sánchez-González L., Fernández-Robles L., Pérez H., Castejón-Limas M. (2019). 3D TDOA Problem Solution with Four Receiving Nodes. Sensors.

[6] Tian Y., Lv J., Tian S., Zhu J., Lu W. (2019). Robust Least-Square Localization Based on Relative Angular Matrix in Wireless Sensor Networks. Sensors.

[7] Wen F. (2019). Computationally Efficient DOA Estimation Algorithm for MIMO Radar with Imperfect Waveforms. IEEE Commun. Lett..

[8] Wen F., Mao C., Zhang G. (2019). Direction finding in MIMO radar with large antenna arrays and nonorthogonal waveforms. Digital Signal Process..

[9] Wu P., Su S., Zuo Z., Guo X., Sun B., Wen X. (2019). Time Difference of Arrival (TDoA) Localization Combining Weighted Least Squares and Firefly Algorithm. Sensors.

[10] Sun Y., Ho K.C., Wan Q. (2019). Solution and Analysis of TDOA Localization of a Near or Distant Source in Closed-Form. IEEE Trans. Signal Process..

[11] Sun Y., Zhang F., Wan Q. (2019). Wireless Sensor Network Based Localization Method Using TDOA Measurements in MPR. IEEE Sensors J..

[12] Yin J., Wang D., Wu Y. (2018). An Efficient Direct Position Determination Method for Multiple Strictly Noncircular Sources. Sensors.

[13] Du J., Wang D., Yu W., Yu H. (2018). Direct Position Determination of Unknown Signals in the Presence of Multipath Propagation. Sensors.

[14] Malanowski M., Kulpa K. (2012). Two Methods for Target Localization in Multistatic Passive Radar. IEEE Trans. Aerosp. Electron. Syst..

[15] Amiot N., Pedersen T., Laaraiedh M., Uguen B. (2012). A Hybrid Positioning Method Based on Hypothesis Testing. IEEE Wirel. Commun. Lett..

[16] Coluccia A., Fascista A. (2018). On the Hybrid TOA/RSS Range Estimation in Wireless Sensor Networks. IEEE Trans. Wirel. Commun..

[17] Shen J., Molisch A.F., Salmi J. (2012). Accurate Passive Location Estimation Using TOA Measurements. IEEE Trans. Wirel. Commun..

[18] Nguyen N.H., Dogancay K. (2016). Optimal Geometry Analysis for Multistatic TOA Localization. IEEE Trans. Signal Process..

[19] Beck A., Stoica P., Li J. (2008). Exact and Approximate Solutions of Source Localization Problems. IEEE Trans. Signal Process..

[20] Chen S., Ho K.C. (2013). Achieving asymptotic efficient performance for squared range and squared range difference localizations. IEEE Trans. Signal Process..

[21] Rui L., Ho K.C. (2014). Elliptic Localization: Performance Study and Optimum Receiver Placement. IEEE Trans. Signal Process..

[22] Coluccia A., Ricciato F., Ricci G. (2014). Positioning Based on Signals of Opportunity. IEEE Commun. Lett..

[23] Wang G., Cai S., Li Y., Jin M. (2014). Second-Order Cone Relaxation for TOA-Based Source Localization with Unknown Start Transmission Time. IEEE Trans. Veh. Technol..

[24] Vaghefi R.M., Buehrer R.M. (2015). Cooperative Joint Synchronization and Localization in Wireless Sensor Networks. IEEE Trans. Signal Process..

[25] Zou Y., Wan Q. (2016). Asynchronous Time-of-Arrival-Based Source Localization with Sensor Positio Uncertainties. IEEE Commun. Lett..

[26] Wang G., Ansari N., Li Y. (2018). A Fractional Programming Method for Target Localization in Asynchronous Networks. IEEE Access.

[27] Kang Y., Wang Q., Wang J., Chen R. (2019). A High-Accuracy TOA-Based Localization Method without Time Synchronization in a Three-Dimensional Space. IEEE Trans. Ind. Informat..

[28] Tomic S., Beko M. (2018). Exact Robust Solution to TW-ToA-Based Target Localization Problem with Clock Imperfections. IEEE Signal Process. Lett..

[29] Wu S., Zhang S., Xu K., Huang D. (2019). Neural Network Localization with TOA Measurements Based on Error Learning and Matching. IEEE Access.

[30] Wei H., Wan Q., Chen Z., Ye S. (2008). A Novel Weighted Multidimensional Scaling Analysis for Time-of-Arrival-Based Mobile Location. IEEE Trans. Signal Process..

[31] Ma Z., Ho K.C. (2014). A study on the effects of sensor position error and the placement of calibration emitter for source localization. IEEE Trans. Wirel. Commun..

[32] Yang L., Ho K.C. (2009). An Approximately Efficient TDOA Localization Algorithm in Closed-Form for Locating Multiple Disjoint Sources with Erroneous Sensor Positions. IEEE Trans. Signal Process..

[33] Ho K.C., Lu X., Kovavisaruch L. (2007). Source Localization Using TDOA and FDOA Measurements in the Presence of Receiver Location Errors: Analysis and Solution. IEEE Trans. Signal Process..

[34] Ma Z., Ho K.C. TOA localization in the presence of random sensor position errors. Proceedings of the IEEE International Conference on Acoustics, Speech and Signal Processing (ICASSP).

[35] Wang Y., Ho K.C. (2015). An Asymptotically Efficient Estimator in Closed-Form for 3-D AOA Localization Using a Sensor Network. IEEE Trans. Wirel. Commun..

[36] Amar A., Leus G., Friedlander B. (2012). Emitter Localization Given Time Delay and Frequency Shift Measurements. IEEE Trans. Aerosp. Electron. Syst..

[37] Jinzhou L., Ho K.C., Fucheng G., Wenli J. Improving the projection method for TOA source localization in the presence of sensor position errors. Proceedings of the 8th Sensor Array and Multichannel Signal Processing Workshop (SAM).

[38] Cao J.-M., Deng B., Ouyang X.-X., Wan Q., Ibrahim Ahmed H., Zou Y. Multidimensional scaling-based passive emitter localization from TOA measurements with sensor position uncertainties. Proceedings of the 13th International Conference on Signal Processing (ICSP).

[39] Amiri R., Behnia F., Noroozi A. (2018). An Efficient Estimator for TDOA-Based Source Localization with Minimum Number of Sensors. IEEE Commun. Lett..

[40] Ho K.C. (2012). Bias Reduction for an Explicit Solution of Source Localization Using TDOA. IEEE Trans. Signal Process..

[41] Kay S.M. (1993). Fundamentals of Statistical Signal Processing: Estimation Theory.

[42] Chan Y.T., Ho K.C. (1994). A simple and efficient estimator for hyperbolic location. IEEE Trans. Signal Process..

[43] Cheung K.W., So H.C., Ma W.K., Chan Y.T. (2006). A Constrained Least Squares Approach to Mobile Positioning: Algorithms and Optimality. Eurasip J. Adv. Signal Process..

[44] Yang K., An J., Bu X., Sun G. (2010). Constrained Total Least-Squares Location Algorithm Using Time-Difference-of-Arrival Measurements. IEEE Trans. Veh. Technol..

[45] Yu H., Huang G., Gao J., Liu B. (2012). An Efficient Constrained Weighted Least Squares Algorithm for Moving Source Location Using TDOA and FDOA Measurements. IEEE Trans. Wirel. Commun..

[46] Lin L., So H.C., Chan F.K., Chan Y.T., Ho K.C. (2013). A new constrained weighted least squares algorithm for TDOA-based localization. Signal Process..

